# Prevalence and Associated Clinical Factors of Tuberculosis Infection and Disease Among Pediatric Household Contacts: A Facility-Based Cross-Sectional Study

**DOI:** 10.7759/cureus.109901

**Published:** 2026-05-29

**Authors:** Mangla Sood, Gulnaz Hasan, Malay Sarkar, Ishaan Sood

**Affiliations:** 1 Pediatrics, Indira Gandhi Medical College Shimla, Shimla, IND; 2 Pulmonary Medicine, Indira Gandhi Medical College Shimla, Shimla, IND; 3 General Medicine, Indira Gandhi Medical College Shimla, Shimla, IND

**Keywords:** active case finding, household contact screening, latent tuberculosis infection, pediatric tuberculosis, tuberculosis preventive treatment

## Abstract

Introduction: Household contact screening is a key strategy for early detection of tuberculosis (TB) disease and infection in children exposed to microbiologically confirmed pulmonary TB. The National Tuberculosis Elimination Program data forms the vital foundation for national policy decisions. Complementary facility-based evaluations remain valuable for capturing region-specific nuances, particularly in geographically challenging areas. This study aimed to estimate the prevalence of TB disease and TB infection among pediatric household contacts and assess associated demographic and clinical factors.

Methods: This facility-based cross-sectional study was conducted at Indira Gandhi Medical College, Shimla, from August 2024 to September 2025. Index cases with microbiologically confirmed pulmonary TB registered on the government of India Nikshay portal were identified, and their household contacts were screened in the pediatric outpatient or inpatient departments as per National Tuberculosis Elimination Program guidance. Clinical assessment, anthropometry, Bacillus Calmette-Guérin (BCG) scar assessment, chest radiography, Mantoux testing, and nucleic acid amplification test-based microbiology were performed as indicated. Associations were assessed using chi-square or Fisher’s exact tests and logistic regression.

Results: A total of 170 pediatric household contacts were evaluated (mean age 9.6 ± 4.9 years; 55.3% male; 62.4% rural). TB disease was diagnosed in 10 contacts (5.9%): six microbiologically confirmed pulmonary TB, two clinically diagnosed pulmonary TB, and two extrapulmonary TB. Among children up to 10 years of age (n=100), the TB disease prevalence was 4.6% (95% CI: 1.8-11.2). Symptomatic presentation was clinically associated with TB disease and anti-tubercular therapy (ATT) initiation (OR: 3.16, 95% CI: 2.05-5.12), whereas age, sex, residence, chullah smoke exposure, and body mass index (BMI) were not statistically significant in unadjusted analysis.

Conclusions: Pediatric household contact screening in routine care yields clinically meaningful TB case detection. A symptom-focused strategy, supported by targeted diagnostics and TB preventive treatment implementation, remains central to pediatric TB control.

## Introduction

Tuberculosis (TB) remains a major cause of morbidity and mortality worldwide, and children continue to bear a disproportionate share of the disease burden in high-incidence settings [1]. Because young children are at a heightened risk of rapid progression from infection to disease, the early identification of exposure and prompt preventive therapy are core pillars of TB control [2]. Household contact screening is therefore emphasized in national and international guidance as a practical, high-yield strategy to detect TB disease and infection among the close contacts of index cases and to initiate timely treatment or preventive therapy [2,3].

Despite this emphasis, important gaps persist in the literature. While the National Tuberculosis Elimination Program (NTEP) generates extensive data to guide national policy, operational knowledge gaps exist for pediatric household contacts in geographically challenging areas. Many studies focus on passive case detection or hospital cohorts, leaving uncertainty about the yield and operational feasibility of systematic contact screening in real-world outpatient services. Furthermore, while the profound impact of specific environmental and physiological risk factors on pediatric TB susceptibility, such as severe malnutrition [4] and indoor air pollution from biomass fuels [5], is well established in the literature, routine programmatic reporting on these critical exposure metrics remains highly inconsistent, making it difficult to compare risk profiles across settings.

Clinical and nutritional vulnerabilities in children, such as malnutrition, comorbidities, and immunosuppression, are recognized risk factors for tuberculosis progression [6]. However, they remain under-characterized in the context of contact investigations. Few studies integrate these variables alongside diagnostic pathways to clarify how they influence screening outcomes. Furthermore, the variability in diagnostic approaches, ranging from symptom-based screening to chest radiography, tuberculin skin testing, and nucleic acid amplification tests (NAATs), complicates interpretation and programmatic decision-making. Given these gaps, there is a need for facility-based evidence describing the profile of pediatric household contacts identified through programmatic contact screening. This includes evaluating exposure characteristics, clinical and nutritional status, diagnostic evaluations, and patient outcomes. Such data can optimize screening algorithm and the implementation of tuberculosis preventive treatment (TPT) within national programs. This study aimed to document household contact screening among children exposed to microbiologically confirmed pulmonary TB in a North Indian hospital. The primary objective was to determine the screening yield of TB infection and active TB disease among evaluated household contacts. The secondary objective was to identify the socioeconomic, clinical, and environmental factors associated with these outcomes.

## Materials and methods

This facility-based cross-sectional study was conducted in the Pediatric Department after approval by the Institutional Ethics Committee (HFW/MC/IEC/2025-65) over one year, from August 2024 to September 2025. Index cases were patients with microbiologically confirmed pulmonary TB who were initiated on anti-tubercular therapy (ATT) under the directly observed treatment, short-course (DOTS) program at the hospital and registered on the government of India Nikshay portal. These index cases (or their caretakers) were contacted telephonically, the study was explained, and verbal willingness to participate was sought. Household contacts of index cases aged below 18 years with documented exposure were asked to attend the pediatric outpatient department (OPD) on any working day for screening, after written informed consent from a parent or guardian (with assent from older children, as appropriate). A household contact was defined as a person who shared the same enclosed living space as the index TB patient for one or more nights, or for frequent or extended daytime periods, during the three months before the initiation of the current TB treatment. Contacts were excluded if they were unable to communicate or attend the OPD despite three reminders, or if they had already initiated ATT or TPT before enrollment.

At enrollment, a structured case record form was used to capture demographic and exposure details, including age, sex, residence, education, and socioeconomic status of the index case, family size, number of contacts, history of smoking or exposure to household biomass fuel (chullah smoke), and comorbidities in the index case or contact. For each child contact, a history of symptoms suggestive of TB (cough, fever, weight loss, decreased appetite, failure to thrive, or other system-specific symptoms), prior TB treatment, and history of immunosuppression were recorded. The physical examination included a general examination, anthropometry (weight, height), body mass index (BMI) or weight-for-age interpretation, and assessment of the Bacillus Calmette-Guérin (BCG) scar. Nutritional status was categorized using age-appropriate standards.

**Figure 1:**
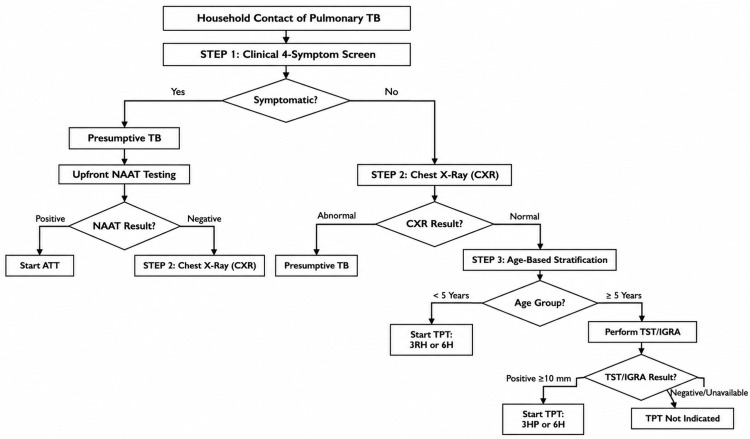
Screening and diagnostic algorithm NAAT: nucleic acid amplification test; ATT: anti-tubercular treatment; TPT: tuberculosis preventive treatment; TST: tubercular skin test; IGRA: interferon-gamma release assay; 3RH: three-month course of daily rifampicin (R) and isoniazid (H); 6H: six-month daily regimen of isoniazid (H)

Investigations were performed as per the NTEP guidelines (Figure 1). All symptomatic contacts and those with risk factors underwent chest radiography. A tuberculin skin test (Mantoux) was performed where indicated. Microbiological testing by NAAT was obtained from sputum when expectoration was possible, or from gastric aspirates in younger children. Additional investigations such as fine-needle aspiration cytology, ultrasonography, or fluid analysis were carried out based on clinical suspicion of extrapulmonary TB. Human immunodeficiency virus (HIV) testing and other relevant investigations were performed as per programmatic protocols when indicated.

Contacts were classified as having TB disease or TB infection based on clinical evaluation, radiology, and microbiological results. Children diagnosed with TB disease were initiated on ATT as per national guidelines. Those with TB infection but without active disease were offered TPT according to NTEP recommendations, with counselling for adherence and follow-up.

Statistical analysis

All data was entered in a dedicated dataset and checked for completeness at the end of each clinic day and complete data was analysed on SPSS version 18 software (SPSS Inc., Chicago, IL, USA). Data was summarized using mean (SD) or median (IQR) for continuous variables and frequency (percentage) for categorical variables. Prevalence of TB infection and disease was estimated, as well as the association with potential determinants assessed using chi-square/Fisher’s exact tests and t-test/Mann-Whitney U test as appropriate; univariable logistic regression was used to evaluate clinical associations with active TB disease. A two-sided p < 0.05 was considered statistically significant.

## Results

**Figure 2:**
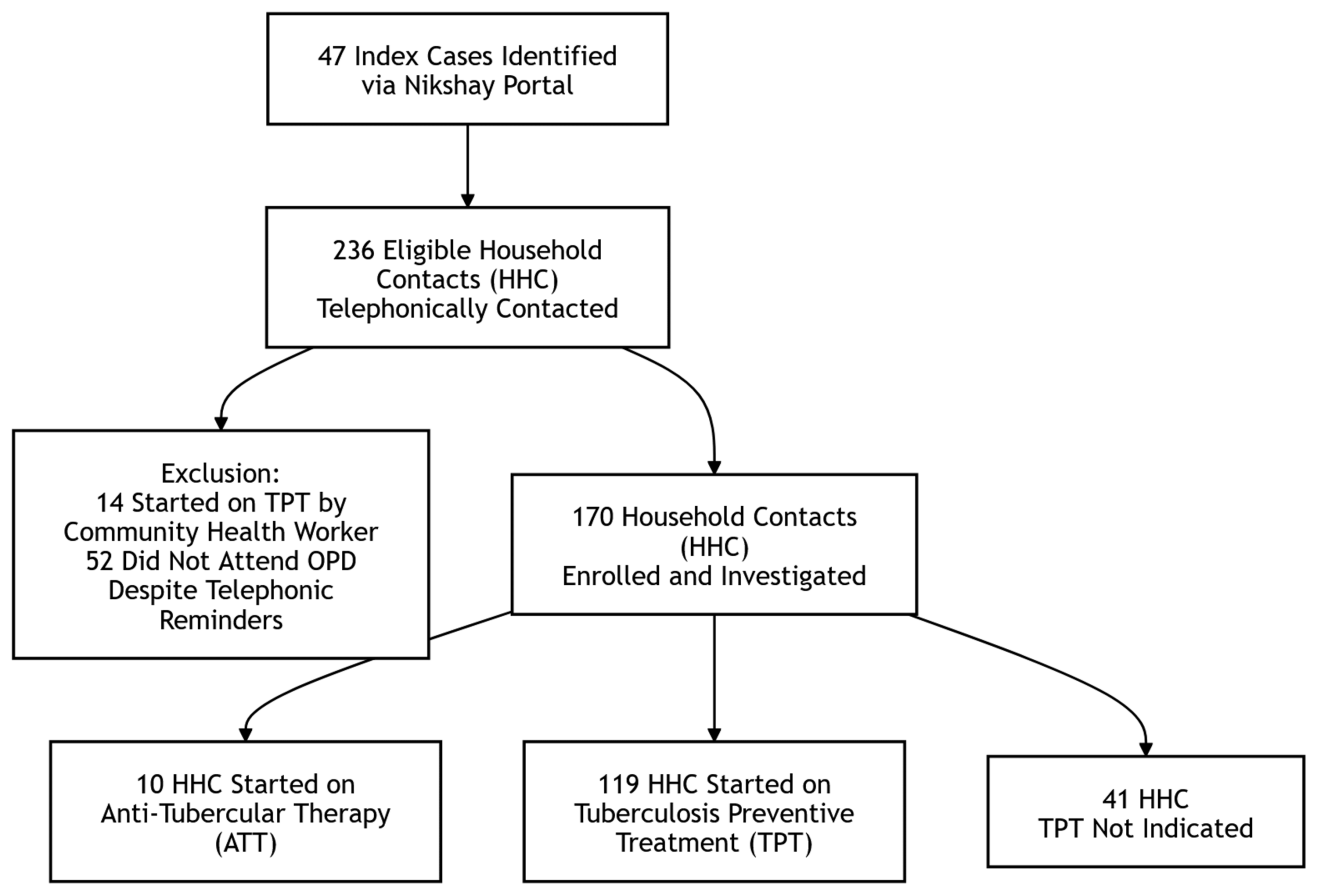
Participant recruitment and clinical outcomes

During the study period, contact tracing was initiated for 47 index cases, identifying a total of 236 eligible pediatric household contacts. Of these eligible children, 170 (72%) agreed to attend the pediatric OPD for systematic clinical screening and were enrolled in the study (Figure 2).

The mean age was 9.6 years (SD 4.9; range 1-18); the cohort was predominantly older, with most patients in the age brackets of older than 10 years (n=70, 41.2%) and over five to 10 years (n=60, 35.3%). There was a slight male predominance overall (n=94, 55.3%), and the majority of contacts resided in rural areas (n=106, 62.4%) (Table 1).

**Table 1:** Baseline characteristics of household contacts (N=170) Values in Number(%); BCG: Bacillus Calmette-Guérin

Characteristic	N(%)
Sex (male)	94 (55.3%)
Residence (rural)	106 (62.4%)
Body Mass Index	
Normal	144 (84.7%)
<-2 SD	11 (6.5%)
-2 to -3 SD	8 (4.7%)
Obese	7 (4.1%)
BCG scar present	167 (98.2%)
Chullah smoke exposure	84 (49.4%)
Symptomatic contacts	10 (5.9%)
Cough duration	
No cough	162 (95.3%)
>4 weeks	4 (2.4%)
2–4 weeks	3 (1.8%)
<2 weeks	1 (0.6%)
Mantoux test	
Negative	108 (63.5%)
Positive	8 (6.9%)
Not done	54 (31.8%)
Chest X-ray	
Normal	162 (95.3%)
Positive	7 (4.1%)
Not done	1(0.6%)
Treatment given	
Preventive therapy	119 (70.0%)
Anti-tubercular drugs	10 (5.9%)
No treatment	41 (24.1%)

Nutritional and immunization status

The overall nutritional status was normal in 144 (84.7%) contacts. A BMI of < -3 SD was observed among 11 children in the age group of one to 10 years, while a BMI between -2 and -3 SD was most frequent in children aged more than one to five years (5/35, 14.3%). BCG vaccination rates were high across the cohort, with a visible scar present in 98.2% of the contacts (Table 2).

**Table 2:** Baseline Characteristics and Nutritional Status Stratified by Age Group Values in Number(%); BMI: Body Mass Index; BCG: Bacillus Calmette-Guérin; *Calculated using Chi-square test; p < 0.05 is significant

Characteristic	≤ 1 year (N=5)	> 1–5 years (N=35)	> 5–10 years (N=60)	> 10 years (N=70)	p-value*
Sex, N (%)					
Male	5 (100.0%)	15 (42.9%)	31 (51.7%)	43 (61.4%)	0.059
Female	0 (0.0%)	20 (57.1%)	29 (48.3%)	27 (38.6%)	
Residence, N (%)					
Rural	2 (40.0%)	25 (71.4%)	40 (66.7%)	39 (55.7%)	0.253
Urban	3 (60.0%)	10 (28.6%)	20 (33.3%)	31 (44.3%)	
BMI, N (%)					
Normal	5 (100.0%)	23 (65.7%)	53 (88.3%)	63 (90.0%)	0.001
-2 to -3 SD	0 (0.0%)	5 (14.3%)	1 (1.7%)	2 (2.9%)	
< -3 SD	0 (0.0%)	7 (20.0%)	4 (6.7%)	0 (0.0%)	
Obese	0 (0.0%)	0 (0.0%)	2 (3.3%)	5 (7.1%)	
BCG Vaccination, N (%)					
Vaccinated	5 (100.0%)	34 (97.1%)	58 (96.7%)	70 (100.0%)	0.468
Unvaccinated	0 (0.0%)	1 (2.9%)	2 (3.3%)	0 (0.0%)	
Chullah Smoke Exposure, N (%)					
Yes	3 (60.0%)	22 (62.9%)	28 (46.7%)	30 (42.9%)	0.249
No	2 (40.0%)	13 (37.1%)	32 (53.3%)	40 (57.1%)	

Clinical profile and tuberculosis diagnostics

Exposure to indoor air pollution via chullahs was reported in nearly half of the cohort (49.4%), with the highest prevalence in children aged five or less (60.0% at one year or less; 62.9% at more than one to five years). Clinically, most household contacts were asymptomatic (94.1%). Active TB disease was diagnosed in 10 patients (5.9%) and was confined to older age cohorts: four children (6.7%) over five to 10 years and six children (8.6%) over 10 years (Table 3).

**Table 3:** Clinical Profile, Diagnostics, and Treatment Outcomes Stratified by Age Group Values in number(%); TB: tuberculosis; ATT: anti-tubercular therapy; *Calculated using Chi-square test, p<0.05 is significant.

Characteristic	≤ 1 year (N=5)	> 1–5 years (N=35)	> 5–10 years (N=60)	> 10 years (N=70)	p-value*
Symptomatic, N (%)					
Yes	0 (0.0%)	0 (0.0%)	5 (8.3%)	5 (7.1%)	0.34
No	5 (100.0%)	35 (100.0%)	55 (91.7%)	65 (92.9%)	
Cough Duration, N (%)					
No cough	5 (100.0%)	35 (100.0%)	57 (95.0%)	65 (92.9%)	0.413
< 2 weeks	0 (0.0%)	0 (0.0%)	1 (1.7%)	0 (0.0%)	
2–4 weeks	0 (0.0%)	0 (0.0%)	2 (3.3%)	1 (1.4%)	
> 4 weeks	0 (0.0%)	0 (0.0%)	0 (0.0%)	4 (5.7%)	
Mantoux, N (%)					
Positive	0 (0.0%)	1 (2.9%)	4 (6.7%)	3 (4.3%)	0.323
Negative	2 (40.0%)	18 (51.4%)	40 (66.7%)	48 (68.6%)	
Not done	3 (60.0%)	16 (45.7%)	16 (26.7%)	19 (27.1%)	
Chest X-Ray, N (%)					
Positive for TB	0 (0.0%)	0 (0.0%)	2 (3.3%)	5 (7.1%)	0.329
Negative for TB	5 (100.0%)	35 (100.0%)	58 (96.7%)	65 (92.9%)	
TB Disease, N (%)					
Yes	0 (0.0%)	0 (0.0%)	4 (6.7%)	6 (8.6%)	0.323
No	5 (100.0%)	35 (100.0%)	56 (93.3%)	64 (91.4%)	
Treatment, N (%)					
Preventive therapy	5 (100.0%)	35 (100.0%)	40 (66.7%)	39 (55.7%)	<0.001
ATT for 6 months	0 (0.0%)	0 (0.0%)	4 (6.7%)	6 (8.6%)	
No treatment	0 (0.0%)	0 (0.0%)	16 (26.6%)	25 (35.7%)	

Treatment and interventions

Seventy percent (n=119) of the cohort was initiated on TPT. Standard ATT for six months was initiated for 10 children, all older than five years, corresponding to active TB diagnoses. No treatment was required for 41 contacts (24.1%).

Univariable regression analysis was performed to evaluate the association between socioeconomic factors and active TB disease among household contacts (Table 4). Due to sparse events, multivariable logistic regression was not attempted.

**Table 4:** Univariable Logistic Regression Analysis of Risk Factors for Starting Anti-Tubercular Treatment *p-value <0.05 is considered significant

Predictor Variable	Odds Ratio (OR)	95% Confidence Interval	p-value*
Symptomatic (Yes vs No)	3.16	2.05-5.12	<0.001
Age (per year increase)	1.16	0.99 – 1.36	0.067
Sex (Male vs Female)	0.34	0.08 – 1.46	0.147
Residence (Rural vs Urban)	0.64	0.11 – 3.63	0.61
Chullah Smoke Exposure (Yes vs No)	0.77	0.13 – 4.73	0.779
Abnormal Body Mass Index (vs Normal)	0.77	0.09 – 6.96	0.819

Age-stratified logistic regression analysis could not be performed because there were zero events in the age groups of under one and one to five years. Among those started on ATT, only symptomatic status was associated with TB disease (odds ratio (OR) 3.16, p = 0.001). The presence of symptoms (such as a cough for over two weeks, fever, or weight loss) was the most frequent clinical marker of disease detection and ATT initiation. The mean age was higher among those with TB disease compared with those without disease (12.4 ± 3.9 vs 9.4 ± 4.9 years; t-test p = 0.043; Mann-Whitney p = 0.056). TB disease did not differ by residence (rural 5/106 vs urban 5/64; Fisher’s exact test p = 0.51) or by sex (female 6/76 vs male 4/94; Fisher’s exact test p = 0.35). Additional factors such as family size (p = 0.14), number of contacts (p = 0.11), and average daily hours of exposure (p = 0.43) were also tested and found to be statistically insignificant. In this specific cohort, risk factors such as malnutrition, indoor air pollution (chullah exposure), and rural living did not statistically increase the risk of developing active TB and requiring ATT. These findings in this cohort support symptom-focused diagnostic evaluation.

## Discussion

This facility-based study described the burden of TB disease and infection among pediatric household contacts of microbiologically confirmed pulmonary TB cases managed under the Nikshay-linked program. The evaluation of 170 pediatric household contacts revealed an overall active TB disease yield of 5.9%. This finding underscores the value of systematic contact evaluation for early identification and aligns closely with global estimates, which typically report a 4% to 5% yield in similar populations. However, the age distribution of active disease in this cohort diverged notably from traditional epidemiological expectations [7-9]. While children under five years of age historically face the highest risk of rapid progression due to immature cellular immunity, all diagnosed cases in this study occurred exclusively in children older than five years. Clinically, symptomatic presentation emerged as the primary indicator associated with active TB disease and ATT initiation, reaffirming the role of symptom-based screening in high-burden settings. Nevertheless, given the majority of contacts were asymptomatic, reflecting the silent nature of early infection, symptom-based screening should be viewed as a primary triage that must be complemented by low thresholds for radiography and microbiological testing where feasible to ensure early case detection in those without clinical signs [2].

Furthermore, the absence of active disease in the under-five cohort provided a crucial window for intervention, and the subsequent 100% TPT initiation. While this represents a highly successful programmatic coverage, the cross-sectional nature of this study limits any assessment of its longitudinal efficacy in preventing future disease progression.

Baseline evaluations revealed a high BCG scar prevalence (98.2%) and a normal nutritional status (84.7%), indicating reasonable overall child health. Nevertheless, a meaningful minority exhibited undernutrition, a well-documented risk factor for disease progression [1].

Regression analysis demonstrated that symptomatic presentation was the primary clinical indicator for the initiation of ATT, while traditional socioeconomic and environmental risk factors did not independently increase the risk in this specific cohort. While established literature strongly links indoor air pollution (specifically biomass cooking smoke) and severe acute malnutrition to increased TB susceptibility [10,11], neither chullah exposure nor an abnormal BMI was statistically associated with active TB in this specific cohort. This lack of statistical association may stem from the high overall prevalence of rural residence (62.4%) and biomass fuel use (48.8%) across the cohort, which restricts their variance as independent analytical variables. Furthermore, the nearly universal BCG vaccination rate and generally normal baseline nutrition may have contributed to a level of individual physiological resilience against these environmental exposures [12-14]. However, these findings must be interpreted with extreme caution. The cross-sectional design and sparse event count preclude any definitive conclusions regarding protective efficacy or causality. The true influence of these environmental and nutritional factors may be more accurately delineated in larger, community-based studies with longitudinal follow-up.

The diagnostic profile also demonstrated a reliance on chest X-ray and NAAT for confirmation, consistent with NTEP guidance, and supports the feasibility of integrating these tools into contact investigations in difficult geographical settings [3]. The observation that no asymptomatic contacts in this cohort were diagnosed with active TB aligns with the World Health Organization and NTEP guidelines, which emphasize that the absence of symptoms is a reliable practical triage criterion to effectively rule out active disease in resource-limited settings.

The strengths of this study included a clearly defined index-case cohort, standardized screening as per national guidelines, and comprehensive capture of exposure and clinical variables. Limitations included its facility-based cross-sectional design, potential selection bias toward contacts who could physically attend the outpatient department, and sparse event counts that constrained multivariable modeling. Furthermore, the diagnostic cascade faced real-world operational hurdles; tubercular skin test was not performed in a portion of the cohort due to intermittent tuberculin stock-outs and the logistical barriers preventing rural families from returning for the 72-hour reading. Additionally, because index cases were drawn from the Nikshay portal, clinical details regarding their disease severity, such as cavitary status or specific smear grading, could not be evaluated. Overall, the findings support sustained household contact screening with a symptom-first approach, complemented by targeted diagnostics [2,3].

## Conclusions

Symptom-based screening remains the vital program tool for identifying active pediatric tuberculosis in household contacts. High uptake of TPT observed in children under five demonstrates that near-complete prophylactic coverage is achievable. To further delineate the association of environmental and nutritional risk factors with TB progression, and to formally evaluate long-term outcomes following TPT initiation, future multicenter, community-based studies with larger sample sizes and longitudinal follow-up are required.
